# Heated-balloon ablation as a novel less invasive local treatment for bile duct strictures: A first experimental study

**DOI:** 10.1371/journal.pone.0322801

**Published:** 2025-05-12

**Authors:** Tadahisa Inoue, Takuya Masuda, Tomoharu Koiso, Mayu Ibusuki, Yusuke Oshima, Yuta Kato, Rena Kitano, Hiromu Kutsumi

**Affiliations:** 1 Department of Gastroenterology, Aichi Medical University, Nagakute, Aichi, Japan; 2 Research & Development Department, Japan Lifeline Co., Ltd., Toda-shi, Saitama, Japan; 3 Medical Research Support Center, University of Fukui Hospital, Fukui, Japan; 4 Department of Gastroenterology, Akashi City Hospital, Akashi, Hyogo, Japan; Tribhuvan University Teaching Hospital, NEPAL

## Abstract

**Background:**

Endobiliary radiofrequency ablation (RF-A) is a promising therapeutic option for bile duct strictures. However, conventional catheter RF-A has several limitations, and its usefulness and application remain debated. This study aimed to examine the feasibility of a novel heated-balloon ablation (HB-A).

**Methods:**

A prototype HB-A catheter, along with an assembly created from freshly resected porcine liver and a specific jig, was used in this study. The test HB-A setting was set in six patterns: 70°C in the target temperature with 2.5 min in the target temperature maintenance time, 70°C with 5 min, 75°C with 2.5 min, 75°C with 5 min, 80°C with 2.5 min, and 80°C with 5 min. The study outcomes included the ablation range and temperature propagation associated with HB-A.

**Results:**

The lengths and depths of the ablation area were 15.8 ± 1.3 and 1.5 ± 0.1, 18.0 ± 0.7 and 2.1 ± 0.1, 18.8 ± 0.4 and 2.3 ± 0.2, 18.5 ± 0.9 and 2.5 ± 0.1, 19.8 ± 0.8 and 2.5 ± 0.1, and 20.5 ± 0.5 and 3.4 ± 0.1 mm in 70°C for 2.5 min, 70°C for 5 min, 75°C for 2.5 min, 75°C for 5 min, 80°C for 2.5 min, and 80°C for 5 min, respectively. Excessive ablation was not observed during any procedure. The temperature cutoff for achieving or not achieving the ablation effect was between 55 and 60°C for every setting.

**Conclusions:**

The novel HB-A treatment has good temperature and ablation range control with high reproducibility under the same settings while preventing excessive ablation. This study paves the way for further evaluation of this procedure and its early clinical application.

## Introduction

Endobiliary radiofrequency ablation (RF-A) via an endoscopic or percutaneous approach is a promising, minimally invasive, local therapeutic option for bile duct strictures [1,2]. RF-A is expected to prolong biliary stent patency and improve survival outcomes, especially in patients with extrahepatic cholangiocarcinoma [3]. However, studies to date on endobiliary RF-A have shown mixed results, with some showing efficacy and others not [4–8], and its usefulness and application remain under debate. Furthermore, problems associated with current RF-A devices, such as bipolar catheters with two or four electrodes at the tip, have been reported. Proper electrode contact with the target tissue is often difficult depending on the lesion, leading to an unstable ablation effect [1,9]. Moreover, the electrode contact area is ablated more strongly than other areas, leading to uneven ablation depth and sometimes adverse events from excessive ablation [10]. Therefore, a breakthrough device that can appropriately manage the temperature and ablation range is essential for the widespread acceptance and development of biliary local ablation therapy.

To overcome this issue, we conceived the concept of balloon catheter-based endobiliary ablation and succeeded in developing the first prototype [10–11]. It is equipped with a balloon at its tip, and eight stretchable electronic inks are printed along the length of the balloon. The balloon structure enables appropriate contact with various forms of strictures through inflation and adjustment of the balloon. However, although the ablation unevenness was significantly lower compared with that of the conventional RF-A device, it remained difficult to eliminate the tendency of the electrode contact area to become severely ablated. For this reason, we further developed a heated-balloon ablation (HB-A) as a novel balloon catheter-based endobiliary ablation, which uses a “hot-water bottle” method in which the electrode does not completely contact the tissue, leading to a good control of ablation effect with safety. This study aimed to examine the feasibility of this novel HB-A treatment and clarify the relationship between ablation setting and range ex vivo using a swine liver model.

## Methods

### Instruments

**Fig 1:**
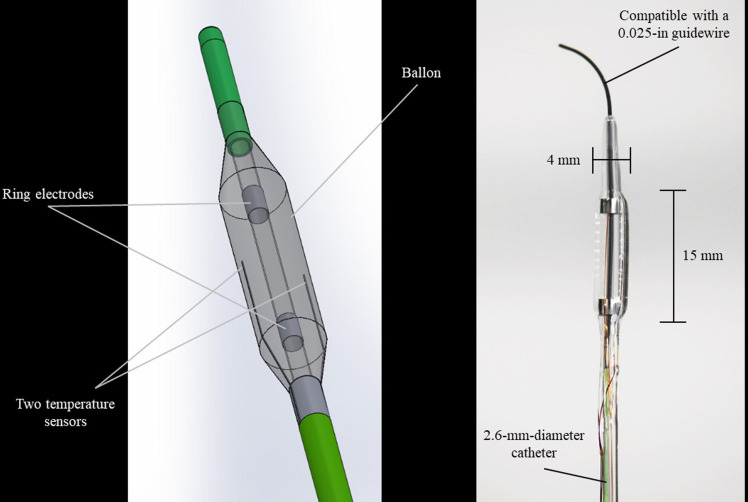
A structural drawing and photograph showing the heated-balloon ablation catheter in an inflated state. The heated-balloon ablation catheter measures 2.6 mm in diameter and 1900 mm in length, which is compatible with a guidewire measuring 0.025-in and can be inserted into a standard duodenoscope working channel. Two 2-mm ring electrodes are located at the tip at a 12-mm interval. A 4-mm-diameter and 15-mm-length balloon is equipped at the tip of the catheter; this balloon is positioned to cover the two electrodes. Two temperature sensors are incorporated on the balloon surface, 180° apart.

The HB-A catheter is shown in Fig 1. It measured 2.6 mm in diameter and 1900 mm in length, which was compatible with a guidewire measuring 0.025 in and could be inserted into a standard duodenoscope working channel. Two 2-mm ring electrodes were placed 12 mm apart at the tip. Furthermore, a 4-mm-diameter and 15-mm-length balloon was equipped at the tip of the catheter; this balloon was positioned to cover the two electrodes. During the HB-A procedure, the balloon was inflated with an ionic contrast agent, and electricity was passed between the two electrodes. Thereafter, the inside of the balloon was gradually heated, and the tissues in contact with the balloon were ablated by conductive heat from the balloon surface. Real-time temperature monitoring can be performed using two temperature sensors incorporated at the balloon surface, 180° apart. Electronic energy was delivered using a modified ARFA-GEN200 generator (Japan Lifeline Co., Ltd., Tokyo, Japan), and the output power was automatically adjusted to maintain the set target temperature.

As the electrode did not come into contact with the tissue and no direct heat production within the tissue was conducted unlike that in conventional RF-A, excessive ablation was suppressed, and the temperature within the tissue and ablation range were easy to control.

### Specimens and preparation

**Fig 2:**
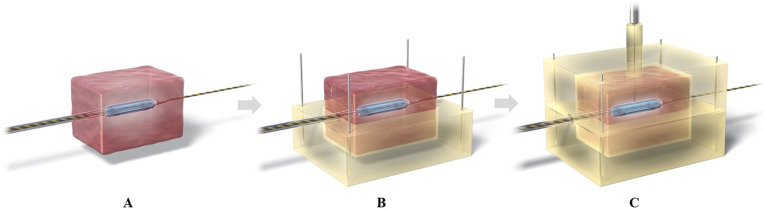
Preparation of experimental setting. Tissue blocks measuring 40 × 40 × 20 mm^3^ were sectioned from porcine liver specimens. A 0.025-in guidewire was passed through its center, followed by insertion of an ablation catheter over the guidewire (A). After the balloon was inflated, this assembly was fitted to the hollowed-out portion of the lower part of jig (B). The assembly was closed with the upper part of the jig, followed by the insertion of the sheath temperature sensor until it approached the balloon surface (C).

**Fig 3:**
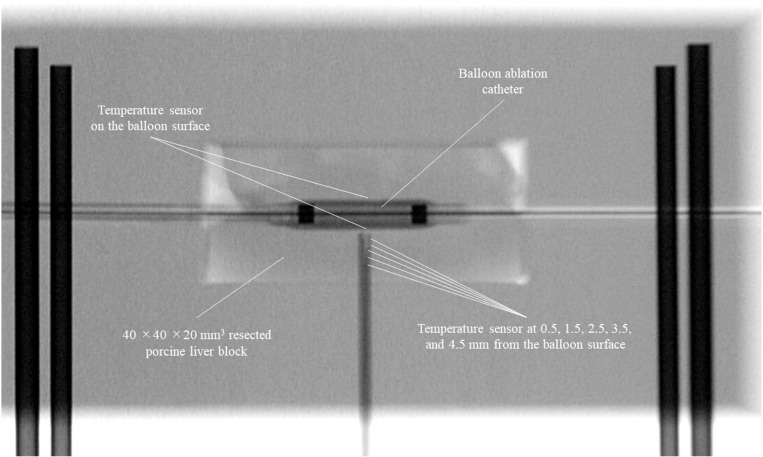
Fluoroscopic view of the experimental setting. The experimental setting was confirmed using fluoroscopy to ensure proper alignment. The sheath temperature sensors were adjusted within the tissue to 0.5, 1.5, 2.5, 3.5, and 4.5 mm from the balloon surface.

Freshly resected porcine liver specimens (TOKYOSHIBAURAZOUKI, Tokyo, Japan) were used for this ex vivo experiment. It was selected for this experiment due to their suitable size and good availability. As no live animals were used, the requirement for ethical approval was waived. First, we developed a specific jig for this experiment. The jig was 100 × 70 × 50 mm^3^ in size with a 40 × 40 × 20 mm^3^ cutout in the center and could be divided into upper and lower parts. Subsequently, blocks measuring 40 × 40 × 20 mm^3^ were sectioned from the porcine liver specimens. A 0.025-in standard guidewire was passed through the center of the block using an inserter, and an HB-A catheter was inserted over the guidewire and positioned at the center of the specimen. After air was sufficiently removed from the inside of the balloon, the balloon was expanded at 4 atm by injecting a contrast agent (Urografin injection 60%, Bayer Yakuhin Co., Ltd., Osaka, Japan). This assembly was fitted into the hollowed-out portion of the jig’s lower part; subsequently, the assembly was closed with the upper part of the jig, followed by the insertion of the sheath-temperature sensors (Fig 2). Five sheath temperature sensors were placed within the tissue at 0.5, 1.5, 2.5, 3.5, and 4.5 mm from the balloon surface. This was confirmed by fluoroscopy to ensure proper alignment (Fig 3). Finally, the assembly was placed in saline solution at 37°C.

### Experimental procedure

The HB-A procedure was initiated at an initial power of 15 W for all the procedures. Once the target temperature was achieved by the balloon surface temperature sensor, the output power was automatically adjusted to maintain the target temperature. The test treatment setting was set in six patterns: 70°C in the target temperature with 2.5 min in the target temperature maintenance time, 70°C with 5 min, 75°C with 2.5 min, 75°C with 5 min, 80°C with 2.5 min, and 80°C with 5 min. Each setting was repeated four times on different specimens. These settings were established based on insights obtained from our previous research [10–12] and preliminary experiments.

After the HB-A procedure, the tissue specimen block was removed from the jig, the balloon was deflated, and the tissue was longitudinally sectioned using a pathology knife along the catheter and perpendicularly sectioned after catheter removal. The ablation range was evaluated and macroscopically measured. The ablated region was fixed in formalin, embedded in paraffin, and sectioned for pathological analysis, which entailed staining with hematoxylin and eosin and Masson’s trichrome to microscopically confirm the ablation effect and range.

### Outcomes and statistical analyses

The study outcomes included the ablation depth associated with HB-A. The ablation depth was defined as the length of the tissue affected by ablation along the direction of the minor axis from the catheter. The temperature propagation around the balloon was also evaluated. Furthermore, the presence or absence of scorching around the balloon, which indicated excessive ablation, was thoroughly evaluated.

Continuous variables are expressed as means and standard deviations. A line graph showing the mean temperature according to depth was created for each setting.

## Results

**Fig 4:**
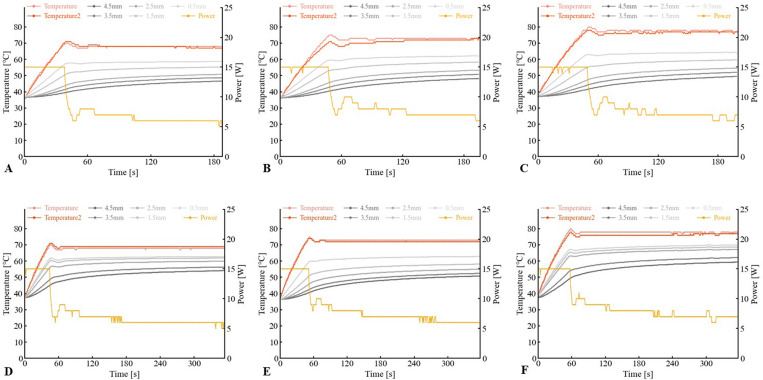
An example of change status in the power and temperature at each point during the heated-balloon ablation. The lines for Temperature and Temperature 2 indicate the transition of the temperature sensor on the balloon surface. Lines of 4.5 mm, 3.5 mm, 2.5 mm, 1.5 mm, and 0.5 mm indicate the temperature measured by the temperature sensor at each point. A, B, C, D, E, and F pertain to 70°C for 2.5 min, 75°C for 2.5 min, 80°C for 2.5 min, 70°C for 5 min, 75°C for 5 min, and 80°C for 5 min, respectively.

**Fig 5:**
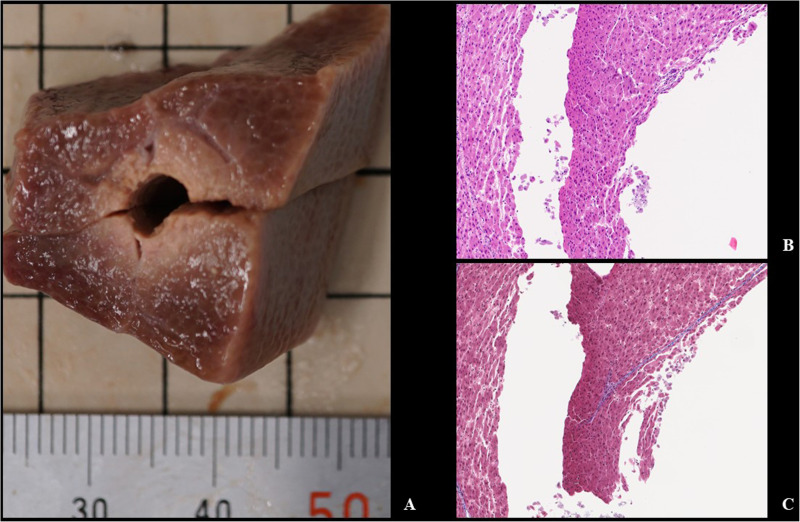
Macroscopic and microscopic findings of the heated-balloon ablation. The ablated area is macroscopically recognized as yellowish-white (A) and histologically recognized as eosinophilic coagulation necrosis with thinning and misalignment of cells (B: hematoxylin and eosin, C: Masson’s trichrome).

Twenty-four specimen samples were created, and all applications of HB-A were technically successful without any hindrance. The experimental results are presented in Table 1 and Supplemental Table1–6 in S1 File. An example of the changes in power and temperature at each point during HB-A for each setting is shown in Fig 4. The target temperature was reached within 60 s in all procedures for all settings. The temperature difference between the two balloon surface temperature sensors, which were located 180° apart, was small when the target temperature was reached, which was within 5°C, for either setting. This value was even smaller at the end of the ablation duration. The ablated area was macroscopically recognized as a yellowish-white change, which was histologically recognized as coagulation necrosis (Fig 5). The lengths of the ablation area, which was the length of the long axis along the catheter, were 15.8 ± 1.3, 18.0 ± 0.7, 18.8 ± 0.4, 18.5 ± 0.9, 19.8 ± 0.8, and 20.5 ± 0.5 mm in 70°C for 2.5 min, 70°C for 5 min, 75°C for 2.5 min, 75°C for 5 min, 80°C for 2.5 min, and 80°C for 5 min, respectively. The depths of the ablation area were 1.5 ± 0.1, 2.1 ± 0.1, 2.3 ± 0.2, 2.5 ± 0.1, 2.5 ± 0.1, and 3.4 ± 0.1 mm, respectively. No events exceeded the target temperature, and no scorching was observed in the tissue around the balloon during any of the procedures.

**Table 1:** Findings of heated-balloon ablation in an animal model.

	70°C for 2.5 min	70°C for 5 min	75°C for 2.5 min	75°C for 5 min	80°C for 2.5 min	80°C for 5 min
Length of ablation area, mm	15.8 ± 1.3	18.0 ± 0.7	18.8 ± 0.4	18.5 ± 0.9	19.8 ± 0.8	20.5 ± 0.5
Depth of ablation area, mm	1.5 ± 0.1	2.1 ± 0.1	2.3 ± 0.2	2.5 ± 0.1	2.5 ± 0.1	3.4 ± 0.1
Time to reach target temperature, s	33.5 ± 3.5	42.5 ± 4.7	43.8 ± 2.7	49.8 ± 5.1	54.0 ± 3.2	53.8 ± 4.3
Difference in temperatures between two temperature sensors on the balloon surface at reaching the target temperature, °C	2.8 ± 1.3	4.3 ± 1.3	3.5 ± 0.5	5 ± 2.5	2.5 ± 0.5	4.8 ± 1.6
Difference in temperatures between two temperature sensors on the balloon surface at finishing the ablation, °C	2.3 ± 0.8	1.8 ± 0.4	1.3 ± 1.3	2 ± 1.2	2.3 ± 0.8	1.5 ± 0.9
Maximum temperature at each temperature sensor, °C						
Balloon surface	70	70	75	75	80	80
Opposite side on the balloon surface	67.8 ± 2.0	66.5 ± 0.5	72.8 ± 0.4	71.8 ± 1.5	77.5 ± 0.5	76.8 ± 1.1
0.5-mm-deep point from the balloon surface	57.8 ± 0.5	59.9 ± 1.3	62.7 ± 1.7	63.6 ± 0.5	65.9 ± 1.5	67.9 ± 1.6
1.5-mm-deep point from the balloon surface	54.5 ± 0.6	56.0 ± 1.7	58.2 ± 1.4	59.7 ± 1.8	61.1 ± 1.9	63.9 ± 3.1
2.5-mm-deep point from the balloon surface	50.7 ± 0.5	53.2 ± 1.7	53.5 ± 1.3	56.6 ± 2.8	56.0 ± 2.0	60.4 ± 4.1
3.5-mm-deep point from the balloon surface	48.5 ± 0.4	51.0 ± 1.6	51.0 ± 1.2	53.8 ± 2.3	53.2 ± 1.7	57.2 ± 3.1
4.5-mm-deep point from the balloon surface	46.6 ± 0.3	49.5 ± 1.4	48.8 ± 1.1	52.0 ± 2.1	50.6 ± 1.6	55.0 ± 2.8

Variables are expressed as the mean±standard deviation.

**Fig 6:**
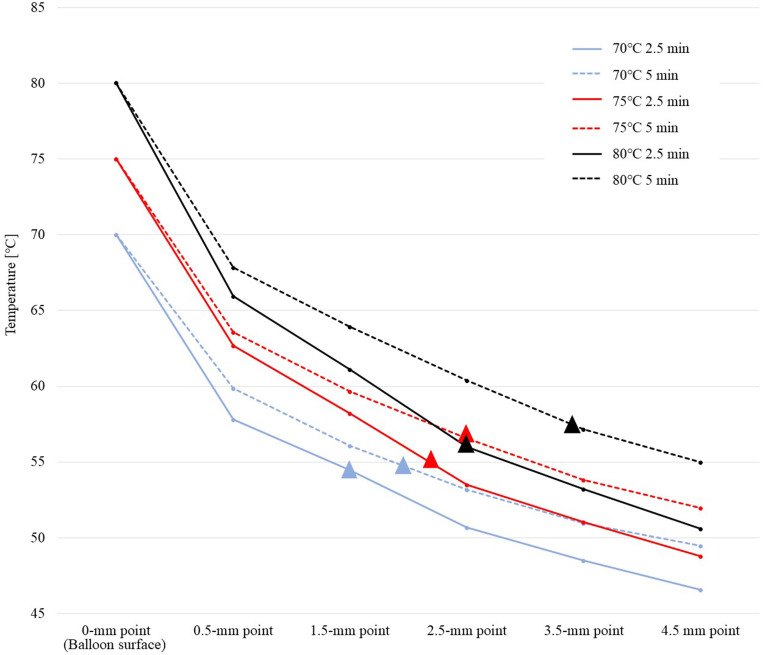
Relationship between the mean maximum temperature within the tissue and distance from the balloon surface. The slopes of the lines for each setting are similar, and the temperature propagation with depth exhibits a similar trend for each target temperature. If the temperature is the same, a longer ablation duration of 5 min results in a higher temperature at each point than those of 2.5-min duration. The point at which the ablation effect was achieved is indicated by a triangle. The boundary between whether or not the ablation effect was achieved was generally between 55 and 60°C.

Fig 6 shows the relationship between the maximum temperature within the tissue and distance from the balloon surface. The point at which the ablation effect was achieved is indicated by a triangle. The slopes of the lines for each setting were similar, and the temperature propagation with depth showed a similar trend for each target temperature. If the target temperature was the same, a longer ablation duration of 5 min resulted in a higher temperature at each point than those of 2.5-min duration. The boundary for achieving the ablation effect was generally between 55 and 60°C for every setting.

## Discussion

This study demonstrates that the novel HB-A has good temperature and ablation range control performance with high reproducibility under the same settings, while preventing excessively and unnecessarily high temperatures. Ablation effect was achieved when the tissue temperature reached approximately 55–60°C or higher, and the ablation depth could be controlled from 1.5 to 3.5 mm using the settings tested in this study.

Endobiliary RF-A is a promising treatment technique for the local treatment of biliary strictures, although effective ablation cannot be achieved in many cases with an existing ablation catheter [1,9]. Currently, biliary RF-A uses a bipolar catheter with two electrodes (or four electrodes) at the tip of the catheter; therefore, it is necessary to properly bring both electrodes into contact with the target lesion. For example, if a lesion is short, non-tight, and soft or has an irregular surface, it is difficult to bring the electrodes into proper contact, resulting in unstable and insufficient effects. In addition, the tissue part that the electrode directly contacted, which means that heat is originally generated within the tissue, is always ablated more strongly than the non-electrode contact area, leading to an uneven ablation depth and effect [10]. Excessive ablation of the electrode contact area can also spread outside the bile duct, leading to adverse events, such as pseudoaneurysm and perforation [13–15]. Therefore, the results of previous studies have been a mixture of positive and negative findings, and this treatment is not widely accepted. To obtain a more stable and safe ablation effect, it is essential to improve ablation devices.

From this point of view, we had developed the balloon RF-A catheter with a stretchable electrode attached to the balloon surface [10–12]. The balloon structure allows for proper contact with the tissue, and a significantly more consistent ablation depth and less excessive ablation were obtained than with the conventional RF-A catheter [10]. However, because the electrodes inevitably come in contact with the tissue, the tendency of the electrode contact portions to be strongly ablated cannot be avoided. Additionally, balloon RF-A is often considered difficult to apply to ingrowth occlusion after metal stent placement, which is another promising indication for endobiliary ablation [16–17]. The clinical success rate of conventional catheter RF-A for ingrowth occlusion is low because it often cannot properly contact the ingrowth tissue because it often has an irregular surface [17]. A balloon structure may be better to solve this problem. However, our previous experiments have shown that the ablation effect is diminished when the electrode comes into contact with the metal stent wire because the current density decreases [18]. Therefore, a balloon RF-A catheter may not be suitable to treat ingrowth occlusion because it is thought to come into contact with the metal stent wire more easily than the conventional catheter.

To overcome these problems, we developed the HB-A treatment as a novel approach for endobiliary ablation. The advantage of HB-A is that while maintaining the benefits of balloon RF-A, which allows contact with any lesion, the electrodes do not come into contact with the tissue, which avoids uncontrollable heat generation within the tissue (this also means preventing contact with the metal stent wire during ingrowth occlusion ablation). Therefore, it is more straightforward to control the temperature within the tissue and ablation range without excessive or uneven ablation spread. In this study, a constant ablation range and good temperature control were obtained with high reproducibility for each setting. Furthermore, the study demonstrated that the ablation depth could be controlled in 1-mm increments, ranging from 1.5 to 3.5 mm. The results of this study show that ablation can be achieved at a tissue temperature of 55–60°C or higher; thus, the ablation depth can be controlled by adjusting the settings to achieve the desired temperature, as indicated by the experimental data. In actual clinical practice, it is possible to measure the desired depth using intraductal ultrasound before ablation, and by setting the ablation conditions according to that value, we can expect to achieve ideal and intended ablation.

The results of this study should be interpreted in the context of its limitations, which arise from its ex vivo experimental design using porcine liver specimens. There is a difference in resistance between healthy and cancerous tissues and human and porcine tissues. Therefore, the settings, including the target temperature and ablation duration, may need to be changed to achieve the same effect in human cancer. Furthermore, the long-term outcomes and adverse events cannot be confirmed in the present study setting. Therefore, the efficacy and safety of this novel treatment must be further investigated in in vivo and clinical studies.

Despite these limitations, this is an important first step toward the development of innovative, minimally invasive, local biliary treatment with promising results and high potential. This basic animal study paves the way for further evaluation of HB-A treatment and its early clinical application.

## Supporting information

S1 File Findings of individual heated-balloon ablations in the animal experiment. (DOCX)
