# Supplementary material for: Heated-balloon ablation as a novel less invasive local treatment for bile duct strictures: A first experimental study
Source: PLoS One. 2025 May 12;20(5):e0322801. doi: 10.1371/journal.pone.0322801 (PMC12068579; doi:10.1371/journal.pone.0322801)
Supplement: S1 File — (DOCX) [file pone.0322801.s001.docx]

**Supplemental Table 1. 70℃ for 2.5 min**

|  | | Specimen 1 | Specimen 2 | Specimen 3 | Specimen 4 |
| --- | --- | --- | --- | --- | --- |
| Length of ablation area, mm | | 14 | 17 | 17 | 15 |
| Depth of ablation area, mm | | 1.4 | 1.5 | 1.5 | 1.5 |
| Time to reach target temperature, s | | 28 | 37 | 36 | 33 |
| Difference in temperatures between two temperature sensors on the balloon surface at reaching the target temperature, ℃ | | 2 | 1 | 4 | 4 |
| Difference in temperatures between two temperature sensors on the balloon surface at finishing the ablation, ℃ | | 2 | 1 | 3 | 3 |
| Maximum temperature at each temperature sensor, ℃ | |  |  |  |  |
|  | Balloon surface | 70 | 70 | 70 | 70 |
|  | Opposite side on the balloon surface | 68 | 71 | 66 | 66 |
|  | 0.5-mm-deep point from the balloon surface | 57.3 | 58.7 | 57.6 | 57.6 |
|  | 1.5-mm-deep point from the balloon surface | 53.6 | 55.3 | 54.6 | 54.4 |
|  | 2.5-mm-deep point from the balloon surface | 49.9 | 50.8 | 51 | 51 |
|  | 3.5-mm-deep point from the balloon surface | 47.8 | 48.8 | 48.7 | 48.7 |
|  | 4.5-mm-deep point from the balloon surface | 46 | 46.7 | 46.8 | 46.8 |

**Supplemental Table 2. 70℃ for 5 min**

|  | | Specimen 1 | Specimen 2 | Specimen 3 | Specimen 4 |
| --- | --- | --- | --- | --- | --- |
| Length of ablation area, mm | | 17 | 19 | 18 | 18 |
| Depth of ablation area, mm | | 2 | 2 | 2 | 2.25 |
| Time to reach target temperature, s | | 50 | 42 | 41 | 37 |
| Difference in temperatures between two temperature sensors on the balloon surface at reaching the target temperature, ℃ | | 3 | 3 | 5 | 6 |
| Difference in temperatures between two temperature sensors on the balloon surface at finishing the ablation, ℃ | | 2 | 1 | 2 | 2 |
| Maximum temperature at each temperature sensor, ℃ | |  |  |  |  |
|  | Balloon surface | 70 | 70 | 70 | 70 |
|  | Opposite side on the balloon surface | 67 | 67 | 66 | 66 |
|  | 0.5-mm-deep point from the balloon surface | 57.7 | 61.2 | 60.5 | 60.1 |
|  | 1.5-mm-deep point from the balloon surface | 53.3 | 57.4 | 57.4 | 56.2 |
|  | 2.5-mm-deep point from the balloon surface | 50.4 | 54.7 | 54.5 | 53.1 |
|  | 3.5-mm-deep point from the balloon surface | 48.3 | 52.2 | 52 | 51.3 |
|  | 4.5-mm-deep point from the balloon surface | 47.1 | 50.7 | 50.3 | 49.8 |

**Supplemental Table 3. 75℃ for 2.5 min**

|  | | Specimen 1 | Specimen 2 | Specimen 3 | Specimen 4 |
| --- | --- | --- | --- | --- | --- |
| Length of ablation area, mm | | 19 | 19 | 19 | 18 |
| Depth of ablation area, mm | | 2.25 | 2.5 | 2.5 | 2 |
| Time to reach target temperature, s | | 42 | 44 | 48 | 41 |
| Difference in temperatures between two temperature sensors on the balloon surface at reaching the target temperature, ℃ | | 3 | 4 | 4 | 3 |
| Difference in temperatures between two temperature sensors on the balloon surface at finishing the ablation, ℃ | | 2 | 0 | 0 | 3 |
| Maximum temperature at each temperature sensor, ℃ | |  |  |  |  |
|  | Balloon surface | 75 | 75 | 75 | 75 |
|  | Opposite side on the balloon surface | 73 | 73 | 73 | 72 |
|  | 0.5-mm-deep point from the balloon surface | 64.2 | 64.2 | 62.2 | 60.1 |
|  | 1.5-mm-deep point from the balloon surface | 59.2 | 59.4 | 58.4 | 55.8 |
|  | 2.5-mm-deep point from the balloon surface | 54.8 | 54.3 | 53.4 | 51.5 |
|  | 3.5-mm-deep point from the balloon surface | 52.3 | 51.8 | 50.8 | 49.2 |
|  | 4.5-mm-deep point from the balloon surface | 50.2 | 49.4 | 48.3 | 47.3 |

**Supplemental Table 4. 75℃ for 5 min**

|  | | Specimen 1 | Specimen 2 | Specimen 3 | Specimen 4 |
| --- | --- | --- | --- | --- | --- |
| Length of ablation area, mm | | 19 | 19 | 17 | 19 |
| Depth of ablation area, mm | | 2.5 | 2.5 | 2.4 | 2.5 |
| Time to reach target temperature, s | | 49 | 56 | 52 | 42 |
| Difference in temperatures between two temperature sensors on the balloon surface at reaching the target temperature, ℃ | | 8 | 6 | 1 | 5 |
| Difference in temperatures between two temperature sensors on the balloon surface at finishing the ablation, ℃ | | 2 | 1 | 1 | 4 |
| Maximum temperature at each temperature sensor, ℃ | |  |  |  |  |
|  | Balloon surface | 75 | 75 | 75 | 75 |
|  | Opposite side on the balloon surface | 71 | 72 | 74 | 70 |
|  | 0.5-mm-deep point from the balloon surface | 63.3 | 64.4 | 63 | 63.5 |
|  | 1.5-mm-deep point from the balloon surface | 58.2 | 59.7 | 58.2 | 62.6 |
|  | 2.5-mm-deep point from the balloon surface | 54.5 | 55.3 | 55.1 | 61.4 |
|  | 3.5-mm-deep point from the balloon surface | 52.1 | 52.8 | 52.6 | 57.8 |
|  | 4.5-mm-deep point from the balloon surface | 50.4 | 51 | 50.9 | 55.6 |

**Supplemental Table 5. 80℃ for 2.5 min**

|  | | Specimen 1 | Specimen 2 | Specimen 3 | Specimen 4 |
| --- | --- | --- | --- | --- | --- |
| Length of ablation area, mm | | 21 | 19 | 19 | 20 |
| Depth of ablation area, mm | | 2.5 | 2.5 | 2.5 | 2.75 |
| Time to reach target temperature, s | | 54 | 53 | 59 | 50 |
| Difference in temperatures between two temperature sensors on the balloon surface at reaching the target temperature, ℃ | | 3 | 2 | 3 | 2 |
| Difference in temperatures between two temperature sensors on the balloon surface at finishing the ablation, ℃ | | 2 | 3 | 3 | 1 |
| Maximum temperature at each temperature sensor, ℃ | |  |  |  |  |
|  | Balloon surface | 80 | 80 | 80 | 80 |
|  | Opposite side on the balloon surface | 77 | 78 | 77 | 78 |
|  | 0.5-mm-deep point from the balloon surface | 68.4 | 65.7 | 65.2 | 64.4 |
|  | 1.5-mm-deep point from the balloon surface | 64.3 | 60 | 60.4 | 59.7 |
|  | 2.5-mm-deep point from the balloon surface | 59.4 | 54.4 | 55.4 | 54.7 |
|  | 3.5-mm-deep point from the balloon surface | 56.1 | 51.7 | 52.9 | 52.1 |
|  | 4.5-mm-deep point from the balloon surface | 53.3 | 49.2 | 50.4 | 49.5 |

**Supplemental Table 6. 80℃ for 5 min**

|  | | Specimen 1 | Specimen 2 | Specimen 3 | Specimen 4 |
| --- | --- | --- | --- | --- | --- |
| Length of ablation area, mm | | 21 | 21 | 20 | 20 |
| Depth of ablation area, mm | | 3.25 | 3.5 | 3.5 | 3.5 |
| Time to reach target temperature, s | | 50 | 59 | 57 | 49 |
| Difference in temperatures between two temperature sensors on the balloon surface at reaching the target temperature, ℃ | | 5 | 6 | 2 | 6 |
| Difference in temperatures between two temperature sensors on the balloon surface at finishing the ablation, ℃ | | 1 | 1 | 1 | 3 |
| Maximum temperature at each temperature sensor, ℃ | |  |  |  |  |
|  | Balloon surface | 80 | 80 | 80 | 80 |
|  | Opposite side on the balloon surface | 77 | 77 | 78 | 75 |
|  | 0.5-mm-deep point from the balloon surface | 66.9 | 66.1 | 70.2 | 68.2 |
|  | 1.5-mm-deep point from the balloon surface | 61.7 | 61 | 68.9 | 64.1 |
|  | 2.5-mm-deep point from the balloon surface | 57 | 57.4 | 67.3 | 59.9 |
|  | 3.5-mm-deep point from the balloon surface | 54.4 | 54.8 | 62.3 | 57.1 |
|  | 4.5-mm-deep point from the balloon surface | 52.6 | 52.9 | 59.6 | 54.8 |
